# Methodological and interpretive concerns about Beemster et al.’s article ‘The interpretation of change score of the pain disability index after vocational rehabilitation is baseline dependent’: a letter to the editor

**DOI:** 10.1186/s12955-020-01555-1

**Published:** 2020-09-07

**Authors:** Laura Wilma Maria Elisabeth Beckers, Rob Johannes Elise Marie Smeets

**Affiliations:** 1 Department of Rehabilitation Medicine, Care and Public Health Research Institute (CAPHRI), Maastricht University, P.O. Box 616, 6200 MD Maastricht, the Netherlands; 2 CIR Revalidatie, Eindhoven, the Netherlands

**Keywords:** Chronic musculoskeletal pain, Pain Disability Index, Minimal important change, Standard error of measurement, Smallest detectable change

## Abstract

This is a critique of Beemster et al.’s article ‘The interpretation of change score of the pain disability index after vocational rehabilitation is baseline dependent’ (2018). The methodological issues in question include the choices of anchor to determine the minimal important change, and the intraclass correlation coefficient on which the calculation of the standard error of measurement was based. We believe these undermine the authors’ interpretation.

## Main text

In their article ‘The interpretation of change score of the pain disability index after vocational rehabilitation is baseline dependent’, Beemster et al. [1] aimed to assess the interpretation of the change score of the Pain Disability Index (PDI) in patients with chronic musculoskeletal pain (CMP) attending vocational rehabilitation. The authors reported that, on the individual level, the smallest detectable change (SDC) was smaller than the minimal important change (MIC), indicating that the PDI can detect a real change in pain-related disability [1]. However, we question this conclusion, given two significant methodological concerns.

First, in our opinion, the choice of the anchor to determine the MIC is unhelpful. A patient-based global perceived effect (GPE) item, i.e. global rating scale (GRS), is generally acceptable to determine the MIC of a patient-reported outcome using an anchor-based method. The GPE consists of a single Likert-scale question by which patients rate, at follow-up, the perceived change in their health status since baseline. A crucial prerequisite is that the GPE assesses the same construct as does the questionnaire under study [2]. While the PDI measures pain-related disability, the GPE item used in this particular study asks patients “to indicate how much their pain has changed since baseline” [1]. The constructs ‘pain’ and ‘pain-related disability’ are very different, especially in patients undergoing chronic pain rehabilitation. Crucially, since pain complaints are often intractable, these interventions specifically aim to decrease pain-related disability. This also applies to the vocational rehabilitation intervention that the study sample underwent [3]. As a result, pain relief is far less common as an intervention success than decreased pain-related disability is. Accordingly, our hypothesis is that the currently used GPE item ‘pain’ might have resulted in less participants who classified themselves as improved (i.e. responders) than if a GPE item ‘pain-related disability’ would have been used. Additionally, it can be assumed that participants who indicated improvements in pain scores perceived a larger reduction of disability than those who did not experience pain relief. Therefore, a more valid GPE (i.e. measuring pain-related disability) would probably have resulted in a smaller change score on the PDI in the group responders and, in turn, have led to a lower MIC.

The second problem with the study is the way the standard error of measurement (SEM) has been calculated. The authors use a formula of debatable validity, SEM = SD √(1-ICC) [1] (where SD is the standard deviation and ICC is the intraclass correlation coefficient). Since they did not perform their own test-retest analysis, their ICC was derived from a study of Soer et al. [4], who extensively validated the PDI in three groups of patients with musculoskeletal pain. Beemster et al. state that this study’s sample was similar to their own [1]. However, comparison of the descriptive statistics does not bear this out. The ICC in the earlier study was calculated in patients with either acute, subacute, or chronic back pain, or chronic widespread pain of the musculoskeletal system [4]. This mixed population (*n* = 845) had a mean baseline PDI score of 37.6 with a SD of 14.6 (personal communication with Soer R.). In contrast, Beemster et al. found a baseline PDI score of 34.7 with a SD of 11.7 in their population of patients with CMP attending vocational rehabilitation [1]. These SDs show the former population to be more heterogeneous than the latter. However, the ICC is highly dependent on heterogeneity [2] and consequently, as de Vet et al. [2] specifically stress, applying a previously reported ICC to a more homogeneous population will lead to SEMs which are too small and therefore misleading. Since the SDCs at the levels of the individual and the group were obtained from these dubious SEMs, we consider these values invalid as well.

In summary, we think that the MIC may be overestimated, whereas the SDC is likely underestimated. Based on the above arguments, we believe that the conclusion that the SDC is smaller than the MIC is questionable. It is quite possible for the SDC and the MIC to be the same or even to be reversed in order of size, as is the case for many outcome measures. If the MIC of the study is in fact smaller than the SDC, change values between the MIC and SDC might be considered clinically important, but not statistically significant, and thus indistinguishable from measurement error. We suggest that a study similar to this one be carried out in the light of the methodological issues raised in this letter, in order to provide higher quality evidence. Without this, the conclusions of the study of Beemster et al. must be approached with caution. Specifically, we would discourage the use of the cutoff scores for clinically relevant changes provided. Aside from the implications for researchers performing responder analyses, health insurance companies, and policy makers, this is particularly relevant to health care professionals seeking to interpret individual change scores in clinical practice.

## Data Availability

Not applicable.

## Abbreviations

CMP: Chronic musculoskeletal pain
GPE: Global perceived effect
GRS: Global rating scale
ICC: Intraclass correlation coefficient
MIC: Minimal important change
PDI: Pain Disability Index
SD: Standard deviation
SDC: Smallest detectable change
SEM: Standard error of measurement
